# Bicarbonate rather than high pH in growth medium induced Fe-deficiency chlorosis in dwarfing rootstock quince A (*Cydonia oblonga* Mill.) but did not impair Fe nutrition of vigorous rootstock *Pyrus betulifolia*

**DOI:** 10.3389/fpls.2023.1237327

**Published:** 2023-08-24

**Authors:** Yanyan Zhao, Yinglong Chen, Songzhong Liu, Fei Li, Mingde Sun, Zhenxu Liang, Zhi Sun, Futong Yu, Zed Rengel, Haigang Li

**Affiliations:** ^1^ Inner Mongolia Key Laboratory of Soil Quality and Nutrient Resources, Key Laboratory of Agricultural Ecological Security and Green Development at Universities of Inner Mongolia Autonomous Region, Inner Mongolia Agricultural University, Hohhot, China; ^2^ Key Laboratory of Agricultural Ecological Security and Green Development at Universities of Inner Mongolia Autonomous Region, Inner Mongolia Agricultural University, Hohhot, China; ^3^ The UWA Institute of Agriculture, & School of Agriculture and Environment, The University of Western Australia, Perth, WA, Australia; ^4^ Institute of Forestry & Pomology, Beijing Academy of Agriculture & Forestry Sciences, Beijing Engineering Research Center for Deciduous Fruit Trees, Key Laboratory of Biology and Genetic Improvement of Horticultural Crops (North China), Ministry of Agriculture, Beijing, China; ^5^ College of Resources and Environmental Sciences, China Agricultural University, Beijing, China; ^6^ Institute for Adriatic Crops and Karst Reclamation, Split, Croatia

**Keywords:** Fe deficiency, bicarbonate, high pH, pear rootstock, quince A, *Pyrus betulifolia*

## Abstract

**Introduction:**

Quince A (*Cydonia oblonga* Mill.), a typical dwarfing rootstock in pear cultivation, is susceptible to iron (Fe) deficiency in calcareous soils. The aim of this study was to compare the strategies in Fe uptake and utilization in dwarfing rootstock quince A (low Fe efficiency) versus a typical vigorous rootstock *Pyrus betulifolia* (PB) with high Fe efficiency.

**Methods:**

Quince A and PB were grown in nutrient solution (pH 6.3) for 4 weeks followed by three pH treatments: pH6.3, pH8.3a (adjusted with hydroxide) and pH8.3b (adjusted with bicarbonate). The Fe uptake and utilization indicators of the rootstocks were assessed at the onset of chlorosis symptoms (after 58 days of treatments).

**Results and discussion:**

In contrast to PB, quince A exhibited Fe deficiency chlorosis under bicarbonate (pH8.3b). Bicarbonate stimulated the root proton secretion, inhibited root growth and ferric chelate reductase (FCR) activity in both PB and quince A, whereas high pH without bicarbonate (pH8.3a) stimulated only root proton release. Both species accumulated more Fe in roots under high pH treatments than under pH6.3, resulting in Fe sufficiency in leaves. Both high pH treatments increased the activity of leaf FCR in PB and quince A. However, extractable Fe(II) concentration in leaves was increased by high pH treatments in PB only. This study demonstrated that depressed Fe(III) reduction in leaves caused by bicarbonate rather than high pH explained Fe deficiency in quince A grown in bicarbonate-containing medium.

## 1 Introduction

Chlorosis caused by iron (Fe) deficiency is common in fruit trees, especially in alkaline and calcareous soils, limiting fruit yield and quality (Abadía et al., 2004; Rombolà and Tagliavini, 2006). Both high pH and bicarbonate in soil solution can lead to Fe deficiency chlorosis of plants in calcareous soils (Lucena, 2000; Valipour et al., 2018). High pH facilitates the hydroxylation of Fe in aerobic conditions and leads to the low availability of Fe in soil (Thomine and Lanquar, 2011). It also limits Fe uptake by inhibiting root growth (Turner et al., 2020). High bicarbonate concentration in soil causes similar effects as high pH with respect to Fe uptake. In addition, bicarbonate impairs the process of Fe reduction at the plasma membrane (Brüggemann et al., 1993; de la Guardia and Alcantara, 1996; Nikolic et al., 2000). It is difficult to distinguish the effects of high pH and high bicarbonate under field conditions (Ding et al., 2020).

Rootstock plays an important role in affecting scion Fe efficiency (Lucena, 2000; Donnini et al., 2009). However, rootstocks differ in resistance to Fe deficiency or responses to low Fe availability (Sahin et al., 2017). For example, root length, root total Fe concentration, and active Fe concentration in roots and leaves were higher in *Pyrus betulifolia* than *Pyrus ussuriensis* under Fe deficiency stress (Xie et al., 2018). Iron-efficient rootstocks of grapevine (Brancadoro et al., 1995; Ksouri et al., 2007) and peach (de la Guardia et al., 1995) improved Fe acquisition through enhancing Fe(III) reduction and rhizosphere acidification under low-Fe stress. The exudation of oxalate and citrate was a strategy of *Malus micromalus* to resist Fe deficiency (Li et al., 2007). In addition, some species and genotypes of fruit trees adapted to acidic soils (e.g. raspberry, blueberry and kiwifruit) may grow in soils with high pH without suffering Fe deficiency, likely due to a capacity to decrease root apoplastic pH (Tagliavini and Rombolá, 2001).

Pear is one of the most important fruit species in China. Most of the trees grown for production are grafted onto vigorous rootstocks. However, dwarfing rootstock is becoming one of the main rootstocks for pear cultivation because it overcomes disadvantages caused by using vigorous rootstock, such as unexpected stock growth and delayed fruiting (Webster, 2002). As a typical dwarfing rootstock of pear, quince A (*Cydonia oblonga* Mill.) is used widely in dwarfing cultivation, but often shows Fe deficiency chlorosis in calcareous soils (Tagliavini and Rombolá, 2001). On the other hand, *Pyrus betulifolia* (PB), a vigorous rootstock widely used in pear cultivation in North China, is known as one of the most resistant rootstocks to Fe deficiency, showing no or mild Fe deficiency symptoms when grown in calcareous soils (Zhao et al., 2020). Pear cultivar ‘Huangguan’ (*Pyrus bretschneideri* Rehd) showed Fe deficiency chlorosis when grafted onto quince A, but not when grafted onto PB in calcareous soil (Zhao et al., 2020). These observations indicated the rootstocks play a primary role in scion Fe efficiency in soils with high pH and high bicarbonate concentration. However, distinguishing the effects of bicarbonate and high pH on Fe uptake and utilization in quince A and PB remains to be done. Therefore, the objectives of this study were to determine a role of bicarbonate and high pH in inducing Fe deficiency chlorosis of quince A and to explore the mechanisms of Fe uptake and utilization in PB grown in high pH/high bicarbonate medium. In this study, we hypothesized 1) bicarbonate rather than high pH would cause Fe deficiency chlorosis in quince A; and 2) PB would have a higher efficiency in Fe uptake, transport and reduction in both roots and shoots than quince A.

## 2 Materials and methods

### 2.1 Plant culture

Woody cuttings of rootstock *Pyrus betulifolia* (PB) and quince A (*Cydonia oblonga* Mill.) with 2 buds were chosen. To promote rooting, one end of the cuttings was dipped in indolebutyric acid solution with a concentration of 10 mM and then quickly inserted into wet sand medium in a greenhouse with a day/night temperature of 35°C/25°C and a relative humidity of 90%/80%. Thirty days later, well-rooted rootstock seedlings of PB and quince A with uniform size (the new shoots were about 5 cm high) were rinsed carefully with deionised water at least three times. Each seedling was fixed in the centre of a foam lid over a 2 L black plastic pot with solution of the following composition (Donnini et al., 2009): 4 mM KNO_3_, 4 mM Ca(NO_3_)_2_, 1.6 mM MgSO_4_, 0.8 mM KH_2_PO_4_, 37 µM H_3_BO_3_, 7.3 µM MnCl_2_, 0.23 µM CuCl_2_, 0.64 µM ZnSO_4_, 0.08 µM (NH_4_)_6_Mo_7_O_24_ and 80 µM Fe-EDTA. The nutrient solution concentration used for the first 2 weeks’ cultivation was one quarter of the above nutrient solution concentration. The initial pH of nutrient solution was adjusted to 6.3 with 0.1 M KOH. The nutrient solution was aerated continuously and replaced every 2 days.

After 4 weeks of growth, seedlings of PB and quince A were transferred to nutrient solutions with three different pH treatments: pH6.3 (adjusted with KOH), pH8.3a (adjusted with 10 mM KOH), and pH8.3b (adjusted with 10 mM KHCO_3_) with five replicates per treatment (Donnini et al., 2009). The pH6.3 and pH8.3a were buffered with both 0.5 mM MES and 0.5 mM TES (Ding et al., 2020). The K_2_SO_4_ was used to balance the potassium supply in all treatments. The treatment solutions were aerated continuously and replaced daily. The pH of treatments pH6.3 and pH8.3a was adjusted by KOH every 8 h. The pH of treatment pH8.3b was adjusted by HCl every 8 h to minimise bicarbonate accumulation.

Plants grew in a phytotron with a day/night regime of 16/8 h, a temperature of 27.2°C/25.4°C and a relative humidity of 78.6%/55.9%. The photosynthetic photon density flux was 200 μmol m^-2^ s^-1^. The 30 pots (one plant per pot) arranged in the phytotron was in a randomized complete block design (six pots per block with five blocks for the five replicates). The plants were harvested after the onset of chlorosis symptoms (after 58 days of treatment).

### 2.2 Determination of shoot height, trunk diameter, leaf area and root morphology

The height and trunk diameter of the new shoots were measured at harvest with a ruler and vernier calipers, respectively. The area of newly expanded fourth leaf (from the shoot tip) on each plant was measured using a leaf area meter (YMJ-B, TOP Instrument, Hangzhou, China). The roots were rinsed carefully with deionised water at least three times, then scanned by a flatbed scanner (MICROTEK MRS-9600TFU2, Shanghai, China) and analyzed in root analysis system (WINRHIZO Pro2004, Quebec, Canada) to obtain the root length and root surface area.

### 2.3 Leaf SPAD and Fe determination

A SPAD-502 meter (Minolta, 144 Osaka, Japan) was used to determine the relative chlorophyll content of the fully expanded fourth leaf from the tip of the shoot. The measured leaves were cut off and rinsed three times with deionised water for Fe concentration determination. The leaves were dried in a forced-draft oven, ground into powder, and digested in 13 M HNO_3_ and 8.8 M H_2_O_2_ at 220°C for 30 min. The total Fe concentration in samples was measured with a plasma emission spectrometer (ICP-OES, OPTIMA 3300 DV, Perkins-Elmer, Waltham, USA). The Fe concentration in roots was determined in the same way.

The extractable Fe(II) concentration in leaves was measured according to the method of Ni et al. (2015) with some modifications. About 0.2 g of fresh leaves was immersed in 10 mL of 1 M HCl for 24 h in the dark. Then, 2 mL *o*-phenanthroline (0.15%, w/v), 2 mL of 3 M NH_4_F and 5 mL of 1 M NaAc buffer were added successively into 1 mL of the leaf extract solution, and constant volume to 25 mL was achieved by adding deionised water. The absorbance was measured at 510 nm by a UV–Vis spectrophotometer (UV1900PC, AuCy Instrument, Shanghai, China) after 30 min.

### 2.4 Determination of ferric chelate reductase activity

The leaf ferric chelate reductase (FCR) activity was measured according to the method of Ojeda et al. (2004) and Sahin et al. (2017). For this purpose, 0.2 g (fresh weight) of leaf samples was rinsed in 5 mL of 0.2 mM CaSO_4_ followed by incubation in 5 mL of reaction solution (10 mM CaSO_4_, 5 mM MES, 0.1 mM Fe(III)-Na-EDTA, 10 mM Bathophenanthrolinedisulfonic acid disodium salt trihydrate; pH 5.5) in the dark at 23°C for 1 h.

The root samples about 15 cm long were cut, rinsed with deionised water, and then dipped into the reaction solution as described above for 30 min (Arakawa and Masaoka, 1997; Zheng et al., 2013). The fresh weight of the roots was recorded after the incubation. The blank solutions without root samples were included as the control. Absorbance was determined at 535 nm by a UV–Vis spectrophotometer (UV1900PC, AuCy Instrument, Shanghai, China). The concentration of reduced Fe was calculated from a standard curve prepared with different concentrations of ammonium ferrous sulfate.

### 2.5 Root-induced pH changes in root-exudate solution

The root proton secretion was measured according to the method of Valipour et al. (2018) with some modifications. Whole plants were removed from the solution 10 h after sunset. Roots were rinsed with distilled water and then immersed in distilled water for 3 h at night. Because intense proton secretion from roots occurs early in the morning, roots were then dipped into 1 L of distilled water (shielded from light) from 5 am to 10 am (the aerial plant part was exposed to light). The pH was measured with a pH-meter (FE20K PLUS PH, Mettler-Toledo, Mettler-Toledo, Switzerland) immediately after the whole plant was removed from distilled water.

### 2.6 Statistical analysis of data

Data were analysed separately for each rootstock using ANOVA. Tukey’s *post-hoc* test (*P* < 0.05) was used to calculate the significant differences among treatments with SPSS 26.0 (Inc, Chicago, USA).

## 3 Results

### 3.1 Plant growth and leaf symptoms

**Figure 1:**
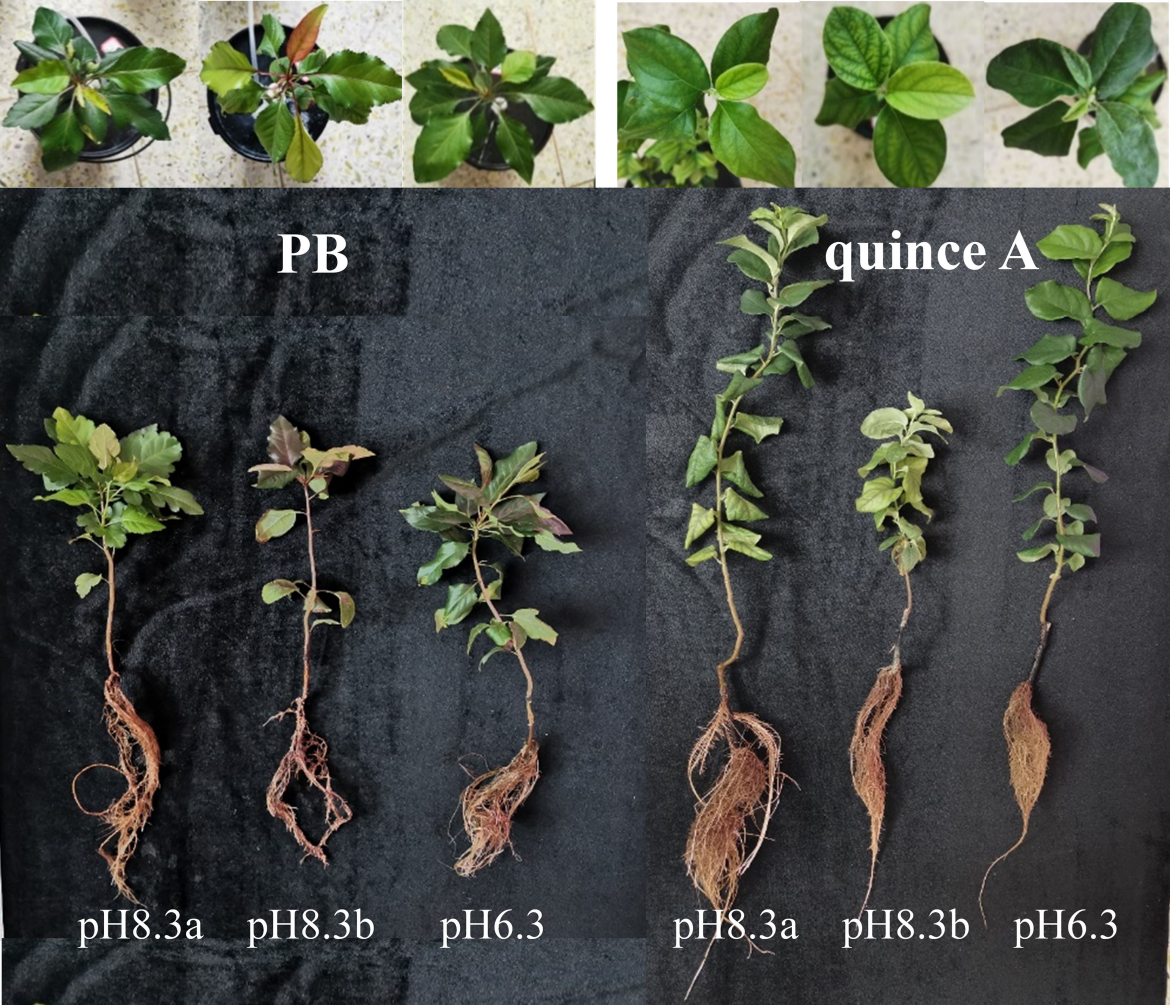
The growth and leaf symptoms of PB and quince A grown in different treatments. The pH6.3 and pH8.3a were adjusted with KOH, whereas pH8.3b was adjusted with KHCO_3_. PB: *Pyrus betulifolia*.

The growth of PB and quince A in pH8.3b was the worst among the three treatments, respectively (**Figure 1**). By contrast, rootstock growth did not differ significantly between the treatments pH8.3a and pH6.3 for both PB and quince A, but the plant height of quince A was greater than that of PB in these two treatments. Bicarbonate addition decreased plant height by 31% in PB and 39% in quince A, trunk diameter by 22% in PB and 31% in quince A, and leaf area by 55% in PB and 45% in quince A compared with pH8.3a (**Figure S1**).

**Figure 2:**
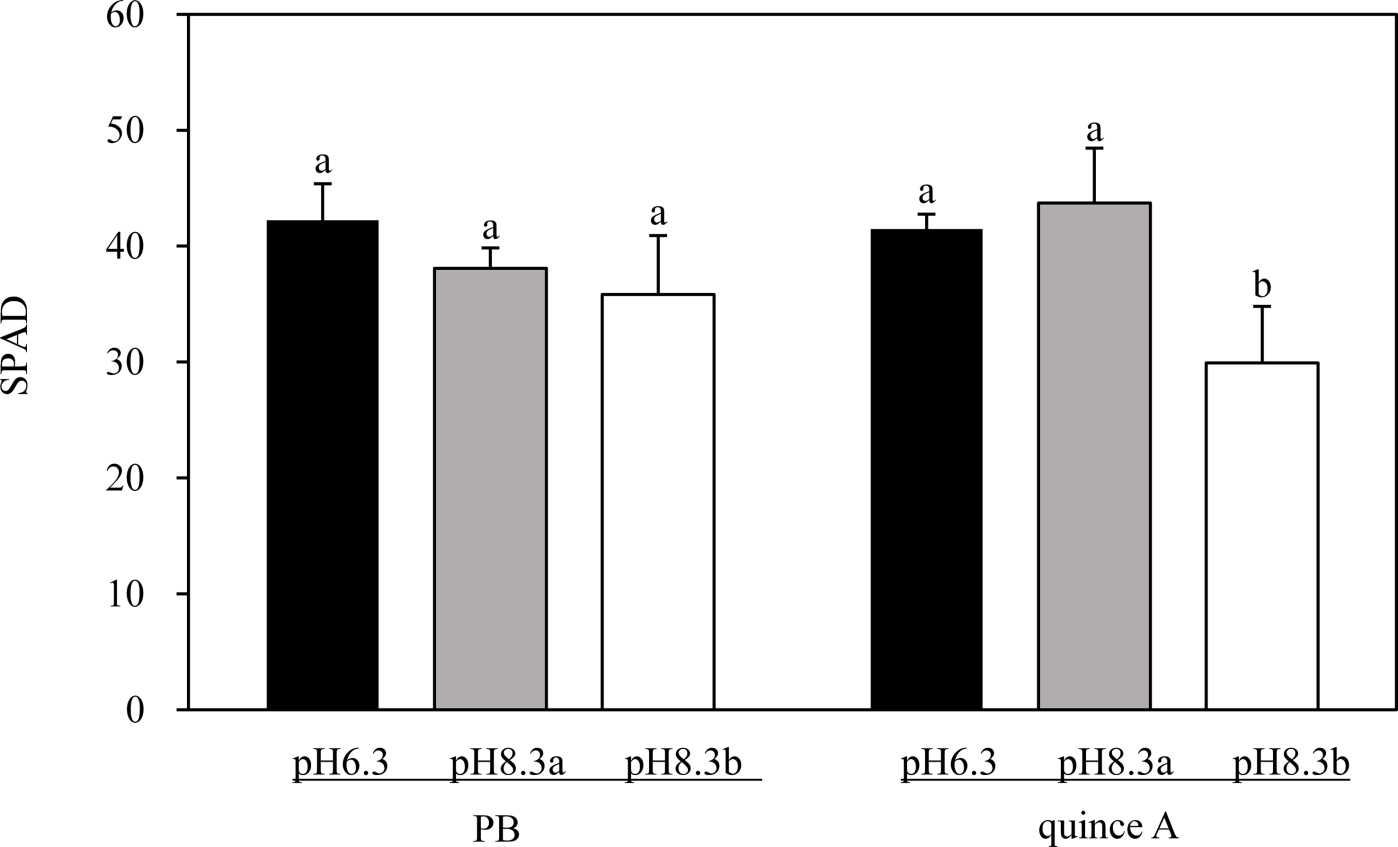
The SPAD of PB and quince A grown in different treatments. The pH6.3 and pH8.3a were adjusted with KOH, whereas pH8.3b was adjusted with KHCO_3_. PB: *Pyrus betulifolia*. Data shown are means + SD (n=5). For each rootstock, different letters indicate significant differences among treatments at *P* < 0.05 (Tukey’s *post-hoc* analysis).

There was no treatment difference in SPAD values of PB (**Figure 2**). By contrast, the leaf chlorosis was observed in quince A in the pH8.3b treatment (**Figure 1**), and the SPAD value in this treatment was about 30% lower than that in pH6.3 and pH8.3a treatments (**Figure 2**).

### 3.2 Root morphological responses

**Figure 3:**
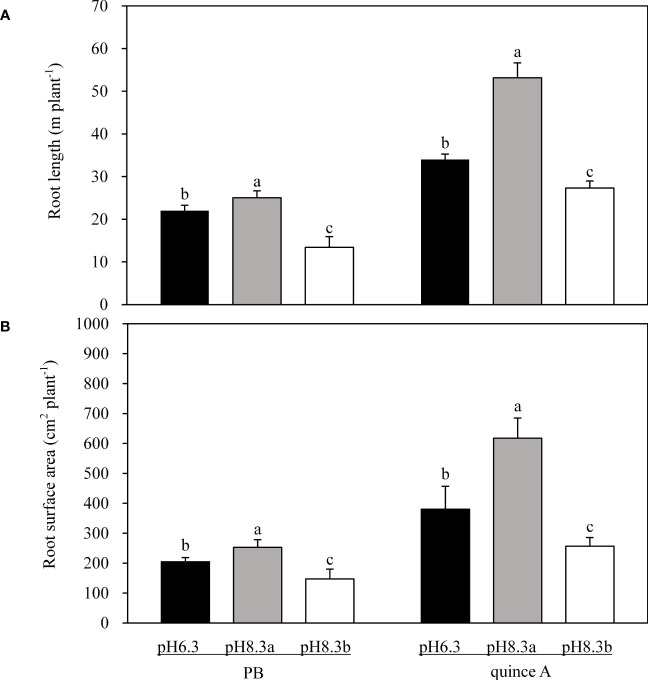
The root length **(A)** and root surface area **(B)** of PB and quince A grown in different treatments. The pH6.3 and pH8.3a were adjusted with KOH, whereas pH8.3b was adjusted with KHCO_3_. PB: *Pyrus betulifolia*. Data shown are means + SD (n=5). For each rootstock, different letters indicate significant differences among treatments at *P* < 0.05 (Tukey’s *post-hoc* analysis).

The root length of both PB and quince A was longest in pH8.3a and shortest in pH8.3b (**Figure 3A**). In the pH8.3b treatment, root length was 54% of that in pH8.3a for PB and 51% for quince A.

The results of root surface areas among the treatments followed the trends of root length (**Figure 3B**). The root surface area of PB was the highest in pH8.3a whilst the lowest in pH8.3b. For quince A, root surface area was the lowest in pH8.3b: 42% and 68% of that in pH8.3a and pH6.3, respectively.

### 3.3 Root physiological responses

**Figure 4:**
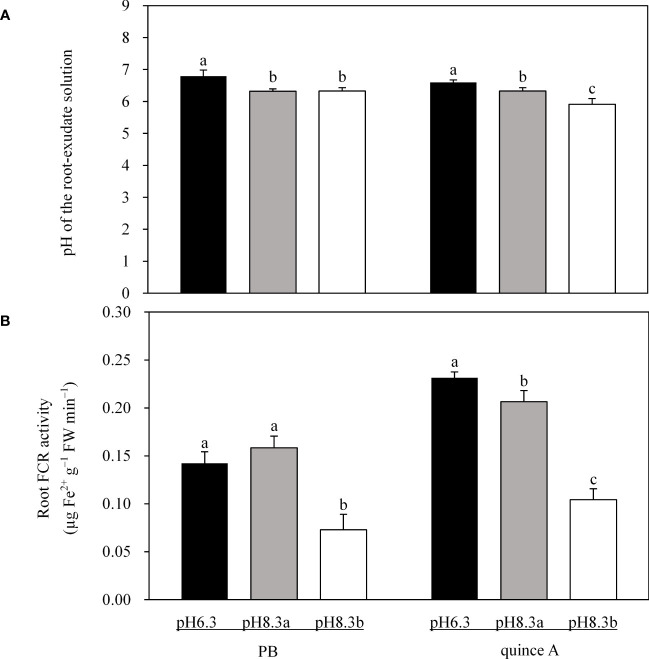
The pH of the root-exudate solution **(A)** and root ferric chelate reductase (FCR) activity **(B)** of PB and quince A grown in different treatments. The pH6.3 and pH8.3a were adjusted with KOH, whereas pH8.3b was adjusted with KHCO_3_. PB: *Pyrus betulifolia*. Data shown are means + SD (n=5). For each rootstock, different letters indicate significant differences among treatments at *P* < 0.05 (Tukey’s *post-hoc* analysis).

The root-exudate solution pH of PB was equal in pH8.3a and pH8.3b treatments (for both 0.40 units lower than that in pH6.3 treatment) (**Figure 4A**). The root-exudate solution pH of quince A decreased by 0.27 and 0.69 units in pH8.3a and pH8.3b, respectively, compared with that in pH6.3 (**Figure 4A**).

Root FCR activity in PB and quince A was about 50% lower in pH8.3b than in pH6.3 and pH8.3a (**Figure 4B**). In quince A, root FCR activity was 8.7% lower in pH8.3a than in pH6.3, but no significant difference between these two treatments was noted in PB.

### 3.4 Fe concentration in roots and leaves

**Figure 5:**
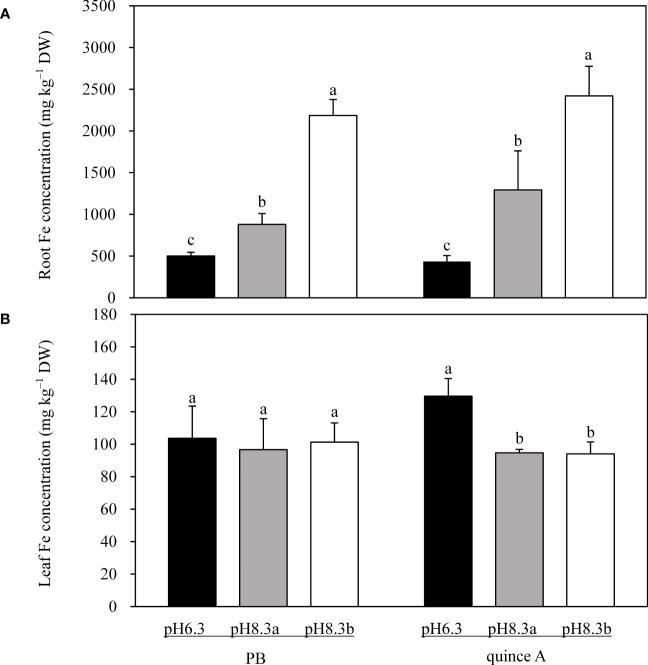
Total Fe concentration in roots **(A)** and leaves **(B)** of PB and quince A grown in different treatments. The pH6.3 and pH8.3a were adjusted with KOH, whereas pH8.3b was adjusted with KHCO_3_. PB: *Pyrus betulifolia*. Data shown are means + SD (n=5). For each rootstock, different letters indicate significant differences among treatments at *P* < 0.05 (Tukey’s *post-hoc* analysis).

Root Fe concentration was highest in pH8.3b and lowest in pH6.3 for both PB and quince A (**Figure 5A**). Root Fe concentration in PB in pH8.3b was 2.5-fold and 4.4-fold higher than that in pH8.3a and pH6.3, respectively. In quince A root Fe concentration in pH8.3b was 2.9-fold and 5.6-fold higher than that in pH8.3a and pH 6.3, respectively.

Leaf Fe concentrations in PB were approximately 100 mg kg^–1^, with no difference among the three treatments (**Figure 5B**). There was no significant difference in leaf Fe concentrations in quince A between pH8.3a and pH8.3b, which were approximately 27% lower than in pH6.3 (**Figure 5B**).

### 3.5 Leaf extractable Fe(II) and leaf FCR

**Figure 6:**
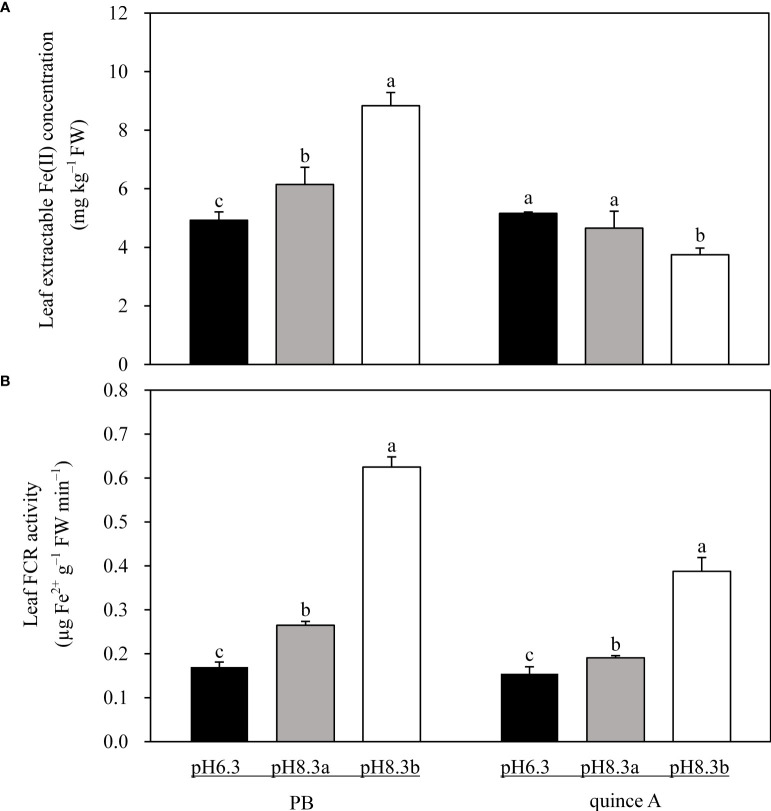
The leaf extractable Fe(II) concentration **(A)** and leaf ferric chelate reductase (FCR) activity **(B)** of PB and quince A grown in different treatments. The pH6.3 and pH8.3a were adjusted with KOH, whereas pH8.3b was adjusted with KHCO_3_. PB: *Pyrus betulifolia*. Data shown are means + SD (n=5). For each rootstock, different letters indicate significant differences among treatments at *P* < 0.05 (Tukey’s *post-hoc* analysis).

In pH8.3b, leaf extractable Fe(II) concentration in PB was 1.8-fold and 1.4-fold higher than that in pH6.3 and pH8.3a, respectively (**Figure 6A**). For quince A, no significant difference in leaf extractable Fe(II) concentration was found between pH6.3 and pH8.3a, and it was approximately 24% lower in pH8.3b (**Figure 6A**).

Leaf FCR activities were higher in the high-pH than low-pH treatments in both rootstocks (**Figure 6B**). In pH8.3b, leaf FCR activity of PB was 3.7-fold and 2.4-fold higher than that in pH6.3 and pH8.3a, respectively. For quince A, compared with pH6.3, leaf FCR activities were 1.3-fold and 2.6-fold higher in pH8.3a and pH8.3b, respectively.

## 4 Discussion

Bicarbonate (treatment pH8.3b) led to Fe deficiency chlorosis in newly expanded leaves in quince A but not in PB. By contrast, no chlorosis symptom was observed in both quince A and PB grown in pH8.3a treatment (high pH but without bicarbonate).

### 4.1 Root responses to pH and bicarbonate

Alteration in root morphology in nutrient-deficient plants is considered as an efficient way to increase nutrients uptake (Reyt et al., 2015). Root length and root surface area in both PB and quince A was significantly increased in pH8.3a compared with pH6.3 (**Figure 3**). However, addition of bicarbonate inhibited root growth of both rootstocks. Bicarbonate is considered as a main factor in inhibiting root growth of some species (Romera et al., 1992; Colla et al., 2012), likely due to bicarbonate inducing Fe deficiency in plants (Yang et al., 1994).

Rhizosphere acidification, as an adaptive strategy of some plants in response to Fe deficiency, facilitates reduction of Fe(III) (Lindsay and Schwab, 1982; Alcantara et al., 1991; Valipour et al., 2018; Zhang et al., 2019). The radical systems by releasing more protons were going to reduce the pH of the solution. The root systems exposed to high pH environments (i.e. pH8.3a and pH8.3b) released more protons to the surrounding media than in low pH (pH6.3) (**Figure 4A**) resulting from the increased H^+^-ATPase activity in the plasma membrane (Tagliavini et al., 1995) because of a huge electrochemical gradient H^+^-ATPase has to work against (Yan et al., 1992). This suggested that the roots of PB and quince A sensed the high pH in the growth medium and might respond by increasing H^+^-ATPase activity. Bicarbonate enhanced proton release in the root system in quince A induced by Fe deficiency in leaves. However, the strong buffering capacity of bicarbonate in the solution of pH8.3b may counteract or diminish the positive effects of rhizosphere acidification.

Root FCR activity is highly related to Fe uptake by Fe-deficient plants (Vert et al., 2002). Bicarbonate inhibits the expression of FCR mRNA in plants such as *Arabidopsis*, pea, tomato and cucumber (Lucena, 2007). Bicarbonate decreased root FCR activities in both PB and quince A (**Figure 4B**). However, de la Guardia and Alcantara (2002) proposed that bicarbonate treatment increased root FCR activity in quince A compared to non-bicarbonate treatment (pH 6.0). Such controversial results may be due to the difference in pH of bicarbonate treatments between the two studies: 7.5 in de la Guardia and Alcantara (2002) and 8.3 in the present study. Lucena (2007) found that the severity of bicarbonate inhibition of FCR mRNA in plants is related to bicarbonate concentration. The high concentration of bicarbonate in this study possibly limited the metabolic activity of the roots in general and also specifically depressed FCR activity. It should be noted that there were no Fe deficiency symptoms in PB leaves in pH8.3b even though root FCR activity was much lower than that in the other two treatments. These results demonstrated that the low value of root FCR activity was sufficient for Fe acquisition to meet PB requirement thanks to its slow growth.

Bicarbonate led to the chlorosis in newly expanded leaves in quince A. However, the restricted root growth and inhibited root FCR activity might not be the main reason for the chlorosis in quince A because total root Fe concentration in bicarbonate treatment (pH8.3b) was higher than that in the other two treatments (**Figure 5A**). Both PB and quince A accumulated Fe in the root systems at pH 8.3, which might be the reason of decreased root FCR activity. This is consistent with the observation of low FCR activity in tomato and cucumber roots with high root Fe concentration (Celletti et al., 2020). The Fe accumulation was not conducive to the transport of Fe to the shoots. High pH in the growth medium may lead to the alkalization of root tissues, especially in bicarbonate conditions (Mengel, 1994), which may promote Fe precipitation inside apoplast in roots, thus causing Fe accumulation (Chaney et al., 1972; Fodor et al., 2012; Wang et al., 2020).

### 4.2 Leaf responses to pH and bicarbonate

It is theoretically feasible to diagnose Fe deficiency chlorosis of fruit trees through leaf nutrient analysis. However, the “Fe chlorosis paradox” always appears in practical production (Morales et al., 1998; Römheld, 2000). This phenomenon also occurred in this study as Fe concentration in the chlorotic quince A leaves in pH8.3b was similar to that in non-chlorotic quince A leaves in pH8.3a (**Figure 5B**). In addition, leaf Fe concentrations in both quince A and PB plants were not less than the threshold of 60 mg kg^–1^ (Jones et al., 1991), suggesting that the amounts of Fe transported from roots to leaves were sufficient even though more Fe accumulated in the roots in pH8.3a and pH8.3b than pH6.3.

Leaf extractable Fe(II) is more reliable than leaf total Fe in assessing Fe nutrition of plants (Pierson and Clark, 1984). Not all Fe in leaves is involved in biological processes because a large proportion of leaf Fe is deposited in apoplasts as inactive Fe (Zhao et al., 2021; Zhao et al., 2023). For quince A, extractable Fe(II) concentration in leaves was significantly lower in pH8.3b than pH8.3a (**Figure 6A**), while the total Fe concentrations were similar (**Figure 5B**). This result demonstrated that more Fe was in hypervalent form in quince A in pH8.3b than pH8.3a, causing Fe deficiency chlorosis in the leaves in pH8.3b. By contrast, PB had a larger proportion of leaf Fe in the Fe(II) form in the presence of bicarbonate (pH8.3b) compared with the other two treatments, suggesting better adaptation of PB to bicarbonate presence than quince A.

The FCR activity is associated with Fe activation as it mediates the reduction of Fe(III) to Fe(II) (Martínez-Cuenca et al., 2017). A decrease in FCR activity has been previously described in leaves of Fe-deficient plants (López-Millán et al., 2001; Sahin et al., 2017). In this study, the increase in FCR activity in leaves of both rootstocks was recorded in high pH treatments, especially in pH8.3b (**Figure 6**). This might be associated with the inhibited root reduction of Fe(III) since the plants took up less Fe and tried to increase Fe uptake from leaf apoplast into mesophyll cells. The result demonstrated that leaf FCR activity was correlated closely with the pH of the growth medium. For PB, extractable Fe(II) in leaves in high pH treatments (**Figure 6A**) increased with the increase of leaf FCR activity (**Figure 6B**), indicating a high FCR activity is essential for Fe reduction. For quince A, leaf extractable Fe(II) was low which induced the increase in leaf FCR activity. However, the leaf chlorosis occurred despite a relatively high leaf FCR activity. Potentially, the available Fe(III), as a substrate for FCR, might have been low in quince A leaves, which limited the reduction.

Lime soil causes pH rise in the root apoplast as well as in the leaf apoplast (Aras et al., 2018). In Fe-deficient leaves of pears, the pH of the apoplast increased from about 0.5 to 1 unit (López-Millán et al., 2001), which might have occurred also in the study presented here. Increased apoplastic pH in leaves would stimulate Fe accumulation by decreasing Fe solubility (López-Millán et al., 2001; Sahin et al., 2017; Zhao et al., 2023). However, Nikolic and Römheld (1999) demonstrates that high bicarbonate supply to the root medium during preculture had no significant effect on the pH of the leaf apoplast. Therefore, the Fe(III) precipitation in the apoplast due to a high apoplastic pH was unlikely a main cause of low Fe(II) in quince A leaves when plants grew in the medium with high bicarbonate concentration. This indicates that there were other factors affecting Fe solubility in addition to the pH, e.g., the forms of Fe compounds with different solubility (Bienfait and Scheffers, 1992). Further studies are needed in the future to supply more solid evidences on this process.

**Figure 7:**
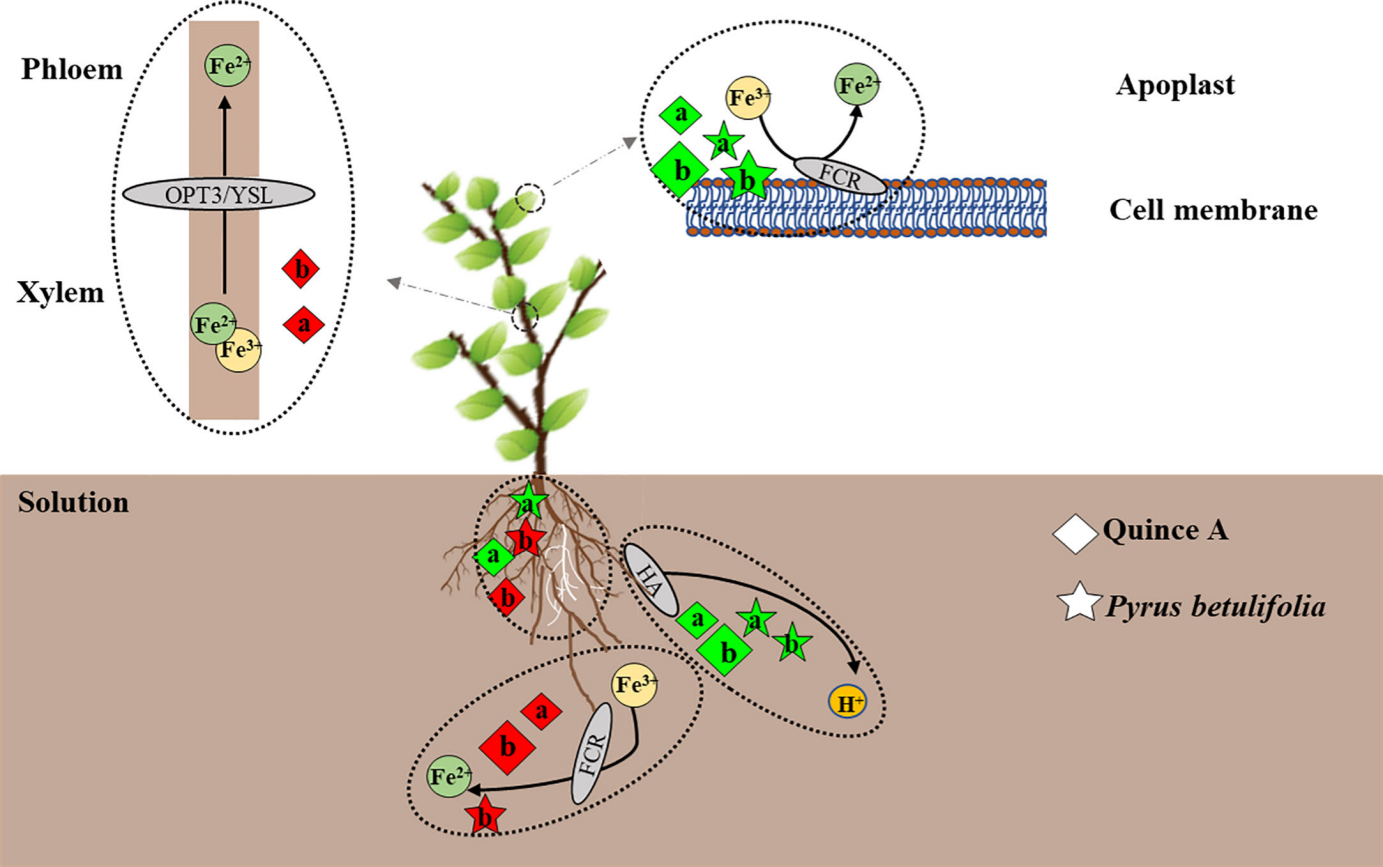
Model of how the solutions of pH8.3a and pH8.3b (without and with bicarbonate, respectively) influence Fe nutrition of *Pyrus betulifolia* and quince A (*Cydonia oblonga* Mill.). The letters “a”, “b” represent the treatments pH8.3a and pH8.3b, respectively. The black arrows indicate the direction of the process. The dotted ovals encircle individual processes. The white roots are the newly grown ones. The diamonds and stars represent quince A and *Pyrus betulifolia*, respectively, with green indicating a positive effect and red a negative effect when compared with the treatment pH6.3. The size of diamonds and stars denotes the relative magnitude of the effect.

In summary, we proposed a model to illustrate Fe uptake, transport and utilization of quince A and PB grown in high pH medium with or without bicarbonate (**Figure 7**). The pH 8.3 without bicarbonate promoted root growth compared with pH 6.3, whereas bicarbonate had an opposite effect. Both high pH treatments stimulated rhizosphere acidification, which could facilitate Fe(III) reduction. Root FCR activity in both quince A and PB was inhibited, and leaf FCR activity was increased, by both high pH treatments. Addition of bicarbonate significantly improved leaf extractable Fe(II) concentration in PB but decreased it in quince A, which was possibly due to low Fe(III) availability in quince A leaves, starving FCR of its substrate. Quince A and PB have similar adaptation strategies to respond to high pH and bicarbonate stresses, but only PB is capable of avoiding Fe deficiency chlorosis due to its higher Fe(III) reduction in leaves compared with quince A.

## 5 Conclusion

Bicarbonate in growth medium significantly inhibited root FCR activity in both PB and quince A, whereas leaf FCR activity was enhanced at pH 8.3 compared with 6.3 in both rootstocks. Bicarbonate increased extractable Fe(II) concentration in leaves of PB and decreased it in quince A. Bicarbonate rather than high pH was responsible for Fe deficiency chlorosis in quince A. This study contributes to the rootstock selections in pear orchards established in calcareous soils.

## Data availability statement

The original contributions presented in the study are included in the article/**Supplementary Material**. Further inquiries can be directed to the corresponding authors.

## Author contributions

YZ organized the data and wrote the first draft of the manuscript. HL, YC and ZR contributed to manuscript revision. SL, HL and YC contributed to the funding acquisition. FY, FL, ZS, MS and ZL contributed to the data curation and supervision. All authors contributed to the article and approved the submitted version.
