# Supplementary material for: Bicarbonate rather than high pH in growth medium induced Fe-deficiency chlorosis in dwarfing rootstock quince A (Cydonia oblonga Mill.) but did not impair Fe nutrition of vigorous rootstock Pyrus betulifolia
Source: Front Plant Sci. 2023 Aug 24;14:1237327. doi: 10.3389/fpls.2023.1237327 (PMC10484346; doi:10.3389/fpls.2023.1237327)
Supplement: Supplementary file 1 [file DataSheet_1.pdf]

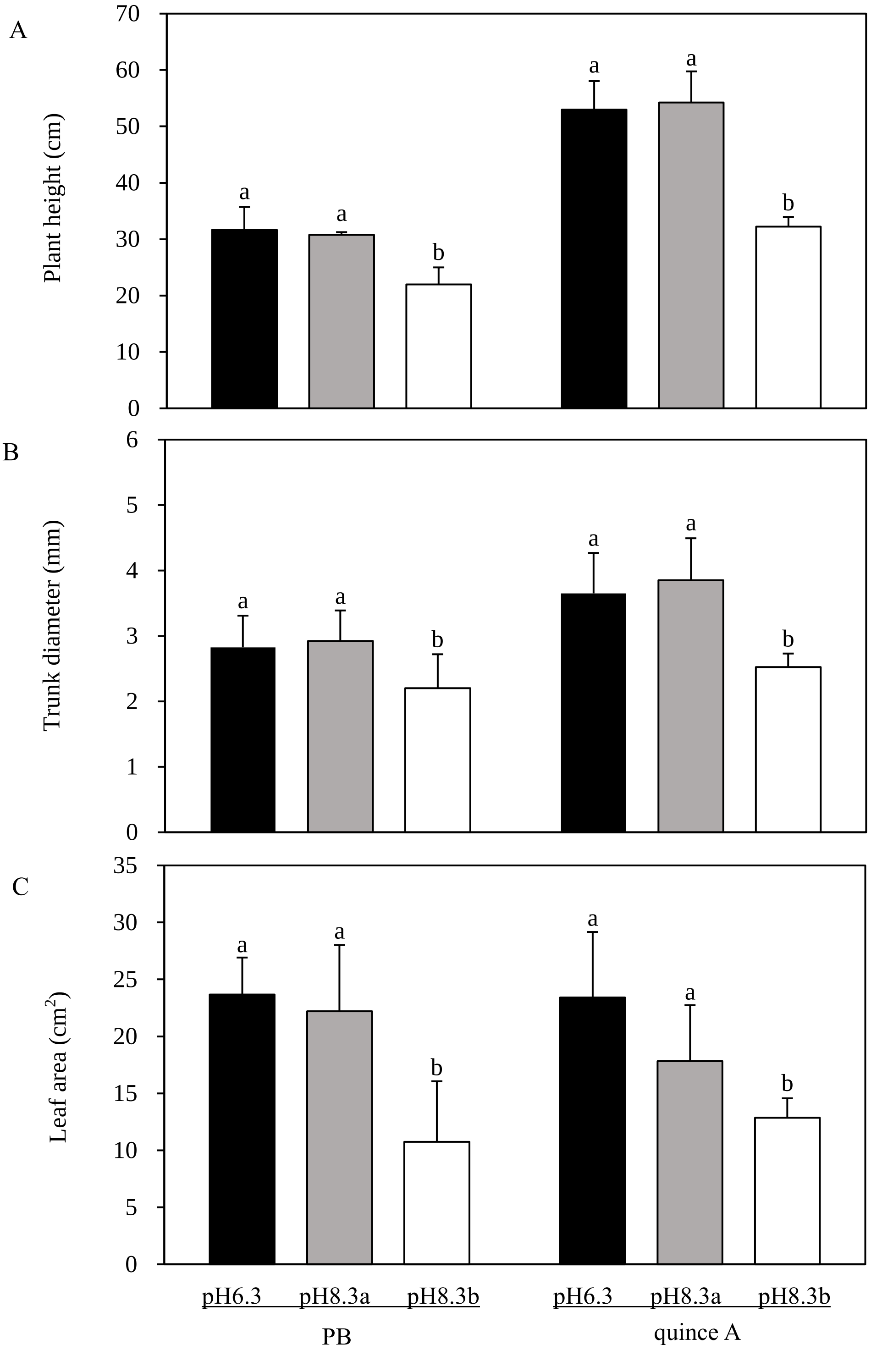

Fig. S1 The plant height (A), trunk diameter (B) and leaf area (C) of PB and quince A grown in different treatments. The pH6.3 and pH8.3a were adjusted with KOH, whereas pH8.3b was adjusted with $KHCO_3$. PB: *Pyrus betulifolia*. Data shown are means + SD (n=5). For each rootstock, different letters indicate significant differences among treatments at $P < 0.05$ (Tukey's post-hoc analysis).
